# Artificial Intelligence Physician Avatars for Patient Education: A Pilot Study

**DOI:** 10.3390/jcm14238595

**Published:** 2025-12-04

**Authors:** Syed Ali Haider, Srinivasagam Prabha, Cesar Abraham Gomez-Cabello, Ariana Genovese, Bernardo Collaco, Nadia Wood, Mark A. Lifson, Sanjay Bagaria, Cui Tao, Antonio Jorge Forte

**Affiliations:** 1 Division of Plastic Surgery, Mayo Clinic, 4500 San Pablo Rd, Jacksonville, FL 32224, USA; 2 Department of Radiology AI IT, Mayo Clinic, Rochester, MN 55905, USA; 3 Center for Digital Health, Mayo Clinic, Rochester, MN 55905, USA; 4 Division of Surgical Oncology, Mayo Clinic, Jacksonville, FL 32224, USA; 5 Department of Artificial Intelligence and Informatics, Mayo Clinic, Jacksonville, FL 32224, USA

**Keywords:** artificial intelligence, generative AI, virtual avatars, digital twins, embodied conversational agents, anthropomorphism, deepfakes, post-operative instructions, patient education

## Abstract

**Background:** Generative AI and synthetic media have enabled realistic human Embodied Conversational Agents (ECAs) or avatars. A subset of this technology replicates faces and voices to create realistic likenesses. When combined with avatars, these methods enable the creation of “digital twins” of physicians, offering patients scalable, 24/7 clinical communication outside the immediate clinical environment. This study evaluated surgical patient perceptions of an AI-generated surgeon avatar for postoperative education. **Methods:** We conducted a pilot feasibility study with 30 plastic surgery patients at Mayo Clinic, USA (July–August 2025). A bespoke interactive surgeon avatar was developed in Python using the HeyGen IV model to reproduce the surgeon’s likeness. Patients interacted with the avatar through natural voice queries, which were mapped to predetermined, pre-recorded video responses covering ten common postoperative topics. Patient perceptions were assessed using validated scales of usability, engagement, trust, eeriness, and realism, supplemented by qualitative feedback. **Results:** The avatar system reliably answered 297 of 300 patient queries (99%). Usability was excellent (mean System Usability Scale score = 87.7 ± 11.5) and engagement high (mean 4.27 ± 0.23). Trust was the highest-rated domain, with all participants (100%) finding the avatar trustworthy and its information believable. Eeriness was minimal (mean = 1.57 ± 0.48), and 96.7% found the avatar visually pleasing. Most participants (86.6%) recognized the avatar as their surgeon, although many still identified it as artificial; voice resemblance was less convincing (70%). Interestingly, participants with prior exposure to deepfakes demonstrated consistently higher acceptance, rating usability, trust, and engagement 5–10% higher than those without prior exposure. Qualitative feedback highlighted clarity, efficiency, and convenience, while noting limitations in realism and conversational scope. **Conclusions:** The AI-generated physician avatar achieved high patient acceptance without triggering uncanny valley effects. Transparency about the synthetic nature of the technology enhanced, rather than diminished, trust. Familiarity with the physician and institutional credibility likely played a key role in the high trust scores observed. When implemented transparently and with appropriate safeguards, synthetic physician avatars may offer a scalable solution for postoperative education while preserving trust in clinical relationships.

## 1. Introduction

### 1.1. Background

When the Lumière brothers screened *Arrival of a Train* in 1896, legend has it that some audience members panicked, fearing the locomotive would burst through the screen [1]. This reaction exemplifies a recurring pattern: technologies that reproduce human experience with unprecedented realism initially provoke eeriness and discomfort until societies adapt [2,3]. Generative artificial intelligence (AI) and synthetic media technologies that replicate human faces, voices, and behaviors represent the newest chapter in this trajectory [4,5].

Deepfakes—videos, images, or audio where a person’s face or voice is digitally altered using deep neural networks—represent a particularly advanced form of synthetic media [6]. These systems can perform face swaps, animate still photographs, or create entirely synthetic people, often with striking realism [7]. While often associated with misinformation concerns, deepfake technology is intrinsically neutral [8] and offers innovative potential for healthcare communication, enabling scalable and personalized patient education [7].

In medical education and clinical practice, AI technologies have demonstrated value across multiple domains. In higher education, AI-powered tools enhance learning through personalized tutoring systems, automated assessment, and adaptive learning platforms [9,10]. In medical education specifically, AI facilitates clinical decision support, diagnostic training through simulation, and standardized patient interactions [10,11]. These applications have shown improved learning outcomes, enhanced student engagement, and more efficient knowledge acquisition compared to traditional methods.

Building on these educational applications, Embodied Conversational Agents (ECAs) or avatars have emerged as a promising interface for AI-mediated healthcare communication [12]. Unlike text-based chatbots, avatars combine visual embodiment with natural speech, gestures, and expressions, making information delivery more engaging and reducing cognitive load [13]. ECAs have been tested in mental health therapy, diabetes education, radiology reporting, and surgical consultation, often showing improved comprehension compared with written formats [12,14,15,16,17].

Virtual physician representations have evolved from simple cartoon characters to sophisticated anthropomorphic avatars [18]. Recent generative AI advances now enable the creation of hyper-realistic “Digital Twins of Doctors” (DTDs)—avatars that closely reproduce a specific physician’s appearance, voice, and speech patterns using deepfake technology [19]. Unlike generic healthcare avatars, DTDs replicate a patient’s actual physician, potentially offering more personalized and scalable education [20]. However, these hyper-realistic representations raise concerns about authenticity, trust, and the “uncanny valley”—a phenomenon where near-human artificial entities evoke feelings of unease or discomfort [18,21].

The COVID-19 pandemic normalized remote digital patient–provider interactions [22,23] and in 2025, the U.S. Centers for Medicare & Medicaid Services highlighted avatars as part of future patient communication strategies [24]. Concurrently, breakthroughs in text-to-video systems such as OpenAI’s Sora and Google’s Veo demonstrate that hyper-realistic videos are becoming increasingly accessible [25,26]. Yet despite this growing momentum, no studies have empirically examined how patients perceive avatars modeled after their own physicians, a critical gap given healthcare’s dependence on trust and the therapeutic relationship

### 1.2. Significance

Understanding patient perceptions of physician avatars is critical because healthcare fundamentally depends on trust, and deploying these technologies without empirical evidence risks undermining therapeutic relationships [19]. Postoperative education represents an ideal use case for avatar-based delivery. Patients frequently forget verbal instructions given immediately after surgery, may hesitate to contact busy clinical teams with routine questions, and often experience dissatisfaction with written discharge materials [27,28,29]. Current paper-based or verbal postoperative instructions are easily forgotten or lost, contribute to unnecessary emergency visits, and place burden on already strained healthcare systems.

### 1.3. Objective

We address this gap through a mixed-methods feasibility study of an interactive surgeon avatar designed for postoperative education, evaluating patient perceptions across usability, engagement, acceptability, trust, realism, and eeriness to provide the first empirical assessment of physician twin avatars in surgical care.

## 2. Methods

### 2.1. Study Design, Setting, and Participants

This pilot feasibility study used an exploratory sequential mixed-methods design to assess patient perceptions of a hyper-realistic AI physician avatar. The study was conducted at Mayo Clinic, Florida, from 11 July to 20 August 2025, and included 30 English-speaking plastic surgery patients who were compensated $100 for their time. Participants consented and provided demographic data, including prior exposure to telehealth, AI chatbots, and deepfakes. To specifically evaluate the unique impact of the “Digital Twin” concept, which leverages patient recognition and established rapport, we selected only patients with a prior clinical relationship with the surgeon. This approach was chosen to assess the specific value of personal familiarity rather than the perception of a generic medical avatar.

### 2.2. Development of the Surgeon Avatar and Study Procedures

We created an interactive AI physician digital twin using the HeyGen IV video generation model (HeyGen Inc., San Francisco, CA, USA) [30], trained on the surgeon’s photographs and a 15-s voice recording. The interaction was orchestrated by a custom, proprietary Python (v3.13)-based software framework developed for this study. To ensure clinical safety and accuracy during this pilot phase, the system was designed to be deterministic rather than generative. We utilized a library of pre-recorded, clinically validated responses covering ten common postoperative topics. During the interaction, user voice input was captured via automated speech recognition (ASR) using Google’s speech-to-text API and processed via Gemini 2.0 flash LLM to identify the question intent for relevant medical topic, triggering the appropriate pre-validated response. This design ensured that all medical advice delivered was strictly controlled by the surgical team, eliminating the risk of AI hallucinations [31,32]. Supplementary Video S1 shows the avatar.

**Figure 1:**
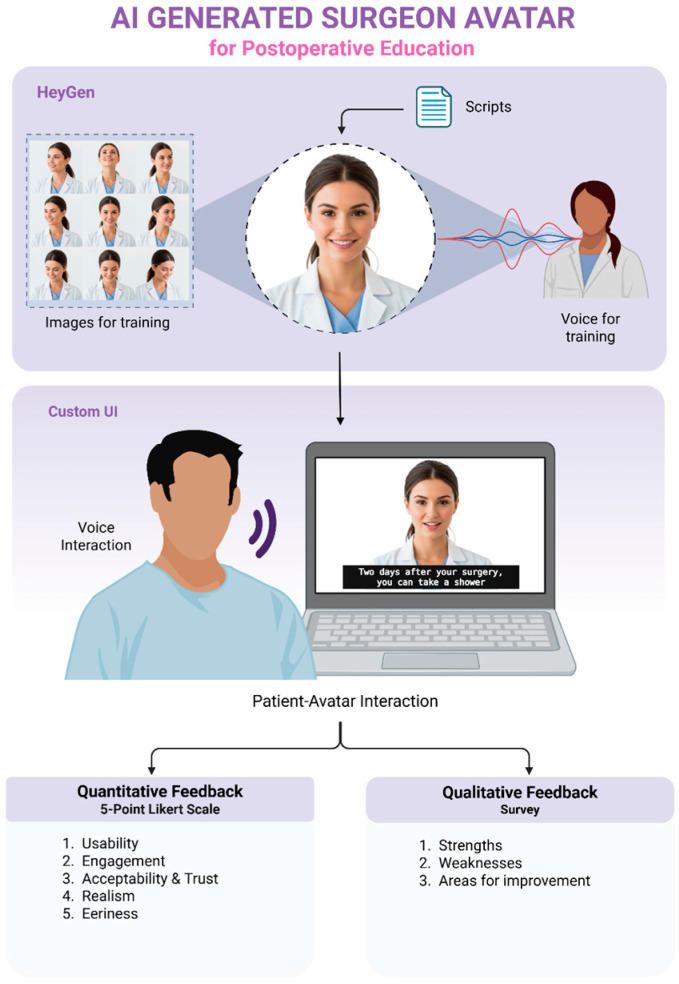
Development and evaluation of an AI-generated surgeon avatar for postoperative education. Created in BioRender. Haider, S. (2025) https://BioRender.com/saa6mbe.

The study took place in a private outpatient room. Participants were provided with a list of ten general postoperative topics (e.g., “Pain Management,” “Drains”) along with optional sample questions for reference. To rigorously test the system’s natural language understanding, participants were explicitly encouraged to create new sentences or modify the samples to match their own phrasing, rather than reading them verbatim. No PHI was collected. If the system failed, participants were allowed one attempt to rephrase the question. System failures were recorded. Each session lasted 8–12 min (3–4 min for interaction, 6–7 min for post-interaction questionnaire). Figure 1 outlines the overview.

### 2.3. Outcome Measures

We developed an assessment battery prioritizing usability, trust, engagement, eeriness, and realism. Domains were assessed using validated scales and context-specific items, with responses on 5-point Likert scales. As a pilot feasibility study, our primary aim was effect size estimation rather than hypothesis testing, with the recognition that the sample size (*n* = 30) limits statistical power for detecting smaller effects across multiple constructs. Negative items were reverse-scored to mitigate response bias.

1. Usability: Measured with the System Usability Scale (SUS) (10 items, scored 0–100; >80 is excellent) [33]. A meta-analysis of digital health apps established a mean benchmark SUS score of 68.05 (SD 14.05) [34].
2. Engagement: Seven items from the User Engagement Scale-Short Form, assessing visual appeal, absorption, and value [17].
3. Acceptability/Trust: Ten items from digital health scales, focusing on trustworthiness, credibility, and recommendation willingness [12].
4. Eeriness/Discomfort: Five items from the uncanny valley literature, assessing unease, visual distortions, and audio-visual mismatch [18].
5. Realism/Human-likeness: Nine items based on anthropomorphism measures and the avatar evaluation literature [18,35], evaluating visual quality, facial stability, movement naturalness, voice quality, and resemblance to the actual physician.

### 2.4. Data Analysis and Ethics

The pilot focused on effect size estimation. We used non-parametric methods (Spearman, Mann–Whitney U, Kruskal–Wallis H) for quantitative analysis. Qualitative data from open-ended feedback was analyzed using Braun and Clarke’s six-phase reflexive thematic analysis. The study was IRB approved (25-002248). Participants were informed they were interacting with an AI replica providing non-medical advice, participation was voluntary, and no PHI was collected.

## 3. Results

### 3.1. Participant Characteristics

**Figure 2:**
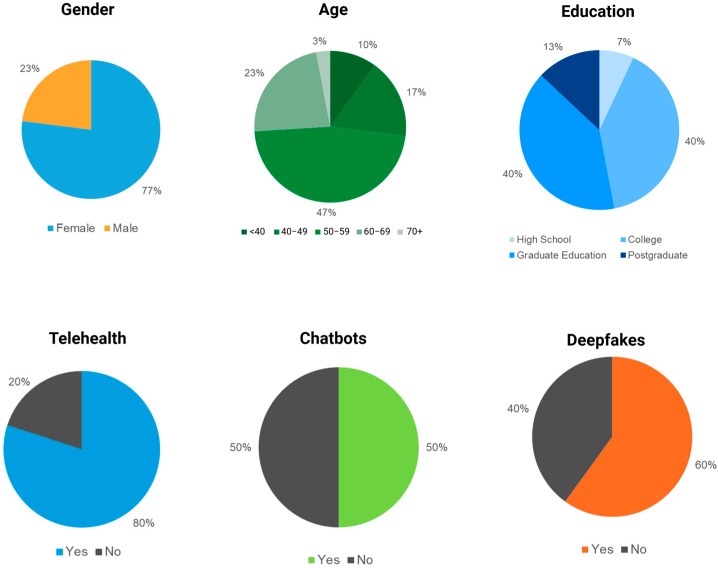
Study Demographics. Created in BioRender. Haider, S. (2025) https://BioRender.com/a4ok256.

Thirty participants (77% female, 23% male) with ages ranging from 33 to 75 years (mean 54.9 ± 9.7 years). Educational attainment was high: 40% had college degrees, 40% had graduate degrees, and 13% held postgraduate degrees. Most participants had prior digital health experience: 80% used telehealth, 50% interacted with AI chatbots, and 60% had seen deepfake videos. The study had a 100% participant retention. Participant demographics are detailed in Figure 2.

### 3.2. Metrics Analysis

**Figure 3:**
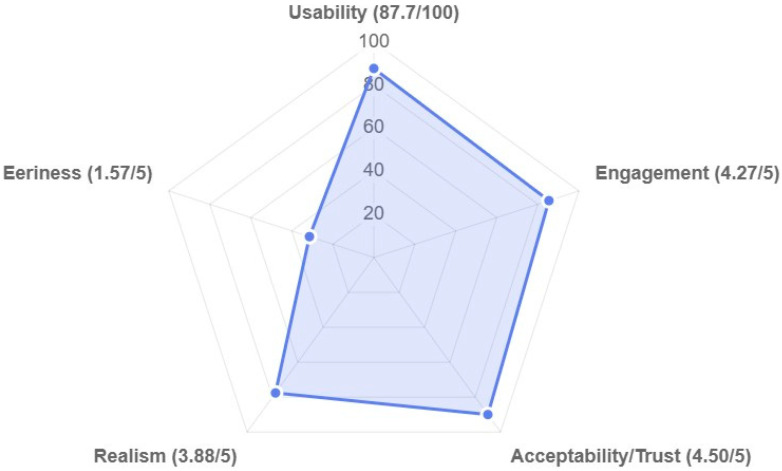
Radar chart depicting overall mean scores for metrics.

The system was highly reliable and stable, answering 99% of patient queries (297/300). Only three failures occurred (one missed, two mismatched responses), none of which affected user experience. Mean domain scores are in Figure 3, with all item-level data in Table 1. Complete descriptive statistics (means, standard deviations) for all items are provided in Table 1. Key findings are summarized below using percentage agreement for interpretability.

For ease of interpretation and to enhance comparability with the existing literature, Likert responses were collapsed into agree (scores 4–5) and disagree (scores 1–2) categories when reporting percentages in the narrative text, while maintaining continuous scores for all statistical analyses. This collapsing approach is well-established in survey research to enhance interpretability, focus analysis on broader trends, and is particularly appropriate for pilot studies with smaller sample sizes [36]. All statistical analyses (correlations, group comparisons) were conducted using the original continuous Likert scale data to preserve measurement precision and statistical rigor.

The system demonstrated excellent usability, achieving a mean SUS score of 87.7 ± 11.5, exceeding the benchmark of 68 for digital health app. Nearly all participants (96.7%) found it easy to use, with 90% reporting confidence. Negative indicators were minimal (10% complexity, 6.7% cumbersome). Participants reported high engagement. Visual appeal was the highest-rated aspect (96.7% agreement), and over 90% found the interaction worthwhile, absorbing, and enjoyable. Temporal immersion (loss of time awareness) was the lowest engagement metric (63.3% agreement). Trust was the highest-scoring domain.

All participants (100%) agreed the avatar was trustworthy and its information believable. Most (96.7%) were satisfied and would recommend the system, finding it highly effective for education. Participants experienced minimal discomfort (low average eeriness score 1.57/5). No one (0%) found the avatar eerie or unsettling. While eeriness ratings were consistently low across all items, facial distortion (M = 1.73, SD = 0.91) and mouth movement (M = 1.63, SD = 0.81) showed modestly greater variability than other eeriness indicators. This suggests that while no participants found these features deeply unsettling, some detected minor technical imperfections in facial rendering and lip-sync quality. Notably, these subtle technical limitations did not translate into overall discomfort or rejection of the technology. This suggests that familiarity with the physician mitigated “uncanny valley” effects. Realism was moderate. While 86.6% recognized the avatar, and technical quality was high, voice matching was less convincing and only 23.3% struggled to distinguish the avatar from a real person, indicating voice was identifiable as artificial.

### 3.3. Correlation Analysis

Usability correlated strongly with engagement (ρ = 0.728, *p* < 0.001), and engagement with trust (ρ = 0.766, *p* < 0.001). These strong positive correlations suggest a cascading acceptance model: when the avatar system is easy to use, patients become more engaged with the interaction; this engagement, in turn, enhances trust in the information provided. This sequential relationship has important implementation implications—ensuring technical usability may be a prerequisite for achieving the engagement and trust necessary for effective patient education.

**Figure 4:**
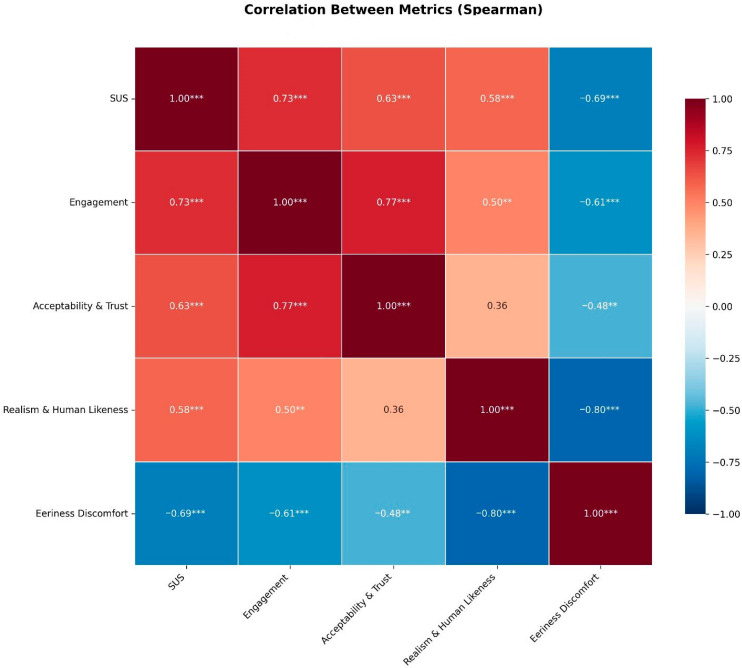
Heatmap showing Spearman correlation between metrics (** *p* < 0.01, *** *p* < 0.001).

Eeriness showed strong negative correlations with realism and usability. This pattern indicates that technical quality serves a dual protective function: higher realism not only makes the avatar more convincing but actively reduces feelings of unease, while better usability prevents frustration that might otherwise manifest as discomfort with the synthetic nature of the interaction. The interrelationship of these domains confirms they are not independent factors but rather interrelated components of overall acceptance (Figure 4).

Participants with prior deepfake exposure (60% of sample) showed a consistent pattern of 5–10% higher acceptance across all domains, though differences were not statistically significant in our small sample. Importantly, deepfake-exposed participants were 26% less likely to believe the avatar was real without disclosure (*p* < 0.05), suggesting familiarity breeds sophisticated acceptance rather than naïve trust. These participants trusted the avatar despite—or perhaps because of—recognizing its synthetic nature, supporting the ‘trust through transparency’ principle discussed in Section 4.3.

No significant associations were found between avatar acceptance and demographic variables (age, gender, education level), nor with prior telehealth or chatbot experience. This suggests that acceptance of physician avatars may be relatively universal across patient demographics within our educated, high-health-literacy sample, though testing in more diverse populations is essential.

### 3.4. Qualitative/Thematic Analysis

Reflexive thematic analysis of open-ended feedback identified five major themes, presented below in order of prominence. Representative quotes illustrate each theme, with complete analysis in Supplementary Materials.

Theme 1: Communication Effectiveness (Most Prominent)

Communication effectiveness emerged as the dominant positive theme, with participants emphasizing both clarity of information delivery and accessibility benefits. Multiple participants highlighted the avatar’s ability to convey medical information comprehensibly: “Easy to understand what he was saying,” “Information explained in easy to understand language,” and “Clarity of verbiage and pace.” Several participants specifically contrasted the avatar favorably with written materials, noting “Voice is easier/quicker to follow than if you were only reading” and “Takes reading comprehension out of the process.” One participant characterized the experience as “So much more engaging than written instructions.” This theme directly validates the quantitative finding that 96.7% found the avatar effective for education, while also highlighting a key advantage: reducing reliance on health literacy and reading comprehension.

Theme 2: Human-Like Interaction Quality

Participants consistently noted the avatar’s success in creating a human-like interaction experience. Comments emphasized both visual realism and the quality of interpersonal connection. Regarding realism, participants observed: “System looks very real as (if) talking with real Dr,” “Very realistic,” and “Very believable as a credible source.” Beyond technical fidelity, participants valued the personal dimension of avatar-based education, describing it as “Human like interaction to provide information more personal than handouts” and noting the avatar “…feels human.” This theme suggests the avatar successfully bridges the gap between impersonal written materials and human interaction, potentially explaining the high engagement scores (M = 4.27).

Theme 3: Technical Limitations

Despite overall positive reception, participants identified specific technical imperfections requiring refinement. Visual artifacts were most commonly noted, including “Slight jilted movements at the end of questions,” “Some small glitches with face,” and “Eyes a little unnatural.” Voice matching emerged as a secondary concern, with several participants noting the avatar “Didn’t sound like my surgeon” and requesting improved “voice alignment.” Interestingly, one participant viewed voice mismatch positively, suggesting individual preferences vary. These technical observations align with the quantitative finding of modest variability in facial distortion (SD = 0.91) and mouth movement (SD = 0.81) ratings. Critically, participants’ identification of these imperfections did not translate into rejection—corroborating our finding that good-enough realism suffices when familiarity mitigates uncanny valley effects.

Theme 4: Content Scope and Personalization

Content limitations emerged as the most frequently cited area for improvement and the primary barrier to expanded utility. Participants consistently requested greater question range, with comments such as “More questions need to be added,” and “Opportunity for more individualized questions.” Procedure-specific information was a particular concern, with participants noting the content “Wasn’t specific to my surgery” and requesting information “More closely related to my procedure.” This theme reflects tension between standardized educational content and individual patient needs. The prominence of this theme suggests that while the avatar interface itself achieved high acceptance, realizing its full potential requires substantial content development and personalization capabilities.

Theme 5: Usability and Accessibility

Participants emphasized the system’s ease of use and immediate accessibility, reinforcing quantitative usability findings (SUS = 87.7). The voice-activated interface was particularly valued: “Voice activated—no key strokes involved” and appreciated for its “Simpleness.” Participants highlighted temporal efficiency and accessibility benefits: “You receive immediate answers,” “Don’t have to wait for someone to respond,” and noting it was a “Good time saver.” These observations validate the system’s design for intuitive interaction and suggest particular value for elderly patients or those with limited digital literacy.

The qualitative themes corroborate and enrich quantitative results. High trust scores (M = 4.60) align with Theme 2’s emphasis on human-like credibility. Minimal eeriness (M = 1.57) despite Theme 3’s technical critiques supports our interpretation that familiarity mitigates imperfection-related discomfort. Most importantly, the prominence of Theme 4 (content scope) suggests that interface acceptance, demonstrated quantitatively is necessary but insufficient for clinical utility; content breadth and personalization remain critical development priorities. A complete thematic analysis with additional quotes appears in Supplementary Materials.

## 4. Discussion

Our pilot study assessed surgical patients’ perceptions of a hyper-realistic avatar of their physician across five domains: usability, engagement, trust, realism, and eeriness. Key findings demonstrated universal trust (all participants found the avatar trustworthy, with 96.7% satisfied or willing to recommend it) and minimal eeriness (mean score 1.57/5). Realism was moderate: 86.5% noted a strong resemblance, though only 50% believed it was real, and it was generally easy to detect as artificial. Overall, patients accepted and trusted the surgeon’s avatar, noting minor issues such as accent mismatch and limited available topics.

### 4.1. Beyond the Uncanny Valley

The “uncanny valley” in avatar research suggests that nearly human but imperfect entities cause discomfort [18,21]. People prefer either stylized or highly realistic avatars, as those in between can seem eerie [37]. The key factor appears to be uncertainty about whether something is human or artificial. Smooth, natural animation also significantly influences positive interaction, often more so than visual detail [35]. For example, Metahuman avatars are consistently rated as the most realistic and socially acceptable in VR [35]. Our pilot findings suggest that clinical avatars may not need perfect realism to avoid eeriness in familiar patient-physician relationship. Patients rated realism as moderately high (mean Likert = 3.88), yet eeriness was very low, and nearly all (96.7%) found the avatar visually pleasing. Therefore, a threshold of “good-enough” realism may suffice, engaging users without unsettling them.

Previous research by Song et al. shows that uncanny avatars erode trust [21]. Coleman et al. found patients distrusted digital clinicians due to limited realism [15]. In contrast to these prior findings, our study observed remarkably low eeriness (M = 1.57) and universal trustworthiness (100%), suggesting our avatar surpassed the perceptual threshold where realism eliminates discomfort. Our perfect trustworthiness scores significantly exceed earlier findings, such as Kim et al.’s 60% [13]. Evidence suggests that AI-generated faces can sometimes be rated as more trustworthy than real ones [38], indicating that once deepfakes achieve sufficient realism they may bridge the uncanny valley, a pattern supported by our findings.

### 4.2. Familiarity as an Antidote to the Uncanny Valley

Research indicates that the uncanny valley effect is lessened by familiarity [39,40]. People tend to favor avatars that represent celebrities [21], actual doctors, or align with their own demographics [13], as these are perceived as less unsettling. This literature also explains our findings: 86.4% of our participants recognized the avatar as their surgeon, and this familiarity likely accounts for the minimal eeriness despite detectable technical imperfections in facial rendering (SD = 0.91) and voice matching (M = 3.83). Custom avatars representing cultural, linguistic, and demographic characteristics of different patient groups can impact comprehension, trust, and engagement across diverse populations [41]. Mori’s original hypothesis and subsequent research support familiarity as an antidote to the uncanny valley, reducing perceived risk and enhancing security [39]. Inconsistent anthropomorphic features and subtle deviations increase unsettling perceptions, but grounding the avatar in a familiar physician may have counteracted this. Additionally, since the study was undertaken at the Mayo Clinic, institutional provenance likely lent legitimacy, boosting trust and acceptance.

### 4.3. Trust Through Transparency

Our study reveals that prior exposure to deepfakes significantly increases acceptance of clinical applications using synthetic media, with participants demonstrating 5–10% higher ratings for usability, trust, and engagement. This challenges the common assumption that awareness of deepfakes breeds skepticism [42]; instead, our findings support the literature, which shows it can foster greater acceptance when the technology is used with consent, without deception, and for positive purposes [8].

Participants with prior deepfake exposure reported greater confidence in distinguishing between real and synthetic content, being 26% less likely to believe an avatar could pass as real without disclosure (*p* < 0.05). However, this likely reflects overconfidence rather than actual detection ability. Existing research consistently shows that people overestimate their deepfake detection capabilities, with actual performance often no better than chance [43]. Our data support this pattern: while deepfake-exposed participants were 26% more confident in detection ability, they still rated the avatar highly trustworthy (M > 4.5), suggesting their confidence did not translate to skepticism when the technology was transparently deployed for beneficial purposes. Studies by Köbis et al. and Chowdhury et al. highlight the “Liar’s Dividend” effect, where humans frequently misidentify genuine videos as fake while failing to detect actual forgeries [43,44]. Our participants likely exhibited similar overconfidence, especially since we explicitly informed them they were interacting with an avatar, and research shows that deepfakes of familiar people are easier to detect than those of strangers [45].

Crucially, when participants believed they could detect deepfakes, their trust levels increased. This points to a preliminary ‘trust through transparency’ principle where individuals are more accepting and less suspicious when they feel capable of identifying synthetic content and understand its appropriate uses. As deepfakes proliferate on social media and become mainstream, increased public familiarity may heighten acceptability, mirroring historical trends where initially unsettling digital technologies eventually gained widespread adoption.

This has significant implications for clinical implementation. Healthcare systems should prioritize transparency in synthetic media and avatar technology as a trust-building strategy. Growing public awareness of synthetic media offers a valuable opportunity for media literacy education, which could foster acceptance of legitimate clinical applications.

### 4.4. Avatars in Healthcare as a Tool

Avatars are already versatile tools in healthcare education, used for tasks like diabetes management [15], postoperative guidance [46], and nuclear medicine training [16]. Research shows patients may prefer avatars over text handouts [13] and pairing avatars with AI-generated voices improves engagement and reduces cognitive load [17]. Furthermore, individuals with social anxiety may find them more approachable than human interactions [47]. Zalake and colleagues developed a physician “digital twin” using Synthesia to study how doctors perceive this technology [19].

While text-based chatbots offer accessible postoperative education [27,28,29], the potential of avatars in this area is largely unexplored. Current verbal or paper-based postoperative instructions often result in patient dissatisfaction [48], are easily forgotten or lost [49,50], and contribute to an overburdened workforce, unnecessary ER visits, and suboptimal outcomes [51]. Avatars address these issues by building on chatbots’ success with added interactivity. Unlike text systems, they combine spoken, visual, and interactive communication [16], making content more accessible and inclusive for patients with literacy, cognitive, age-related, or language barriers [7]. By reducing reliance on reading comprehension, avatars make educational material more memorable, engaging, and personalized.

Avatars also offer scalability and efficiency. A single digital doctor twin can reach thousands of patients across various platforms, delivering consistent, standardized instructions while reducing the time and cost of producing materials. In our study, 93% of participants found the avatar interaction worth their time, valuing the immediate answers. Crucially, patients viewed avatars as a useful supplement, not a replacement, for physicians. Their utility is greatest in delivering standardized, low-stakes education (e.g., FAQs, discharge instructions). This frees up physician time for building rapport and addressing complex or emotionally sensitive decision-making [52], which still requires a human clinician.

### 4.5. Limitations and Strengths

This pilot feasibility study has several limitations. The restricted sample size (n = 30) and single-center design limit generalizability. Participant demographics were not representative—predominantly female (77%) and highly educated (80% with college or graduate degrees)—which may not reflect diverse socioeconomic backgrounds or health literacy levels. All participants were existing patients with prior relationships to the surgeon, an intentional design choice to evaluate the unique “Digital Twin” concept leveraging familiarity and established rapport, but this limits generalizability to first-time encounters with unfamiliar physicians’ avatars.

The brief interaction (10 questions, 8–12 min) mirrors actual postoperative patient–provider phone consultations, which are typically short, discrete encounters. However, this duration does not capture potential longer-term uncanny valley effects or conversational complexity of real clinical dialog.

The avatar system used pre-recorded responses rather than real-time AI generation—a deliberate safety-first methodology choice for this pilot study to eliminate hallucinations and ensure clinical accuracy. This prioritized clinical safety over conversational flexibility; future iterations will integrate retrieval-augmented generation as safety protocols mature. The speech recognition performance was not independently evaluated; real-world deployment across diverse populations and settings may yield different results. Finally, participant compensation ($100) may have introduced positivity bias, and a Hawthorne effect cannot be excluded [53].

Key strengths include the use of real surgical patients and its pioneering role as the first empirical evaluation of a hyper-realistic interactive physician digital twin developed using synthetic media. Another strength is the use of a systematic, multi-domain evaluation (usability, engagement, trust, eeriness, realism) for comprehensive assessment.

### 4.6. Future Research

To address the limitations of this pilot study, future research must prioritize larger, multi-center trials involving more diverse patient populations. Given that our cohort was predominantly female (77%) and highly educated (80%), it is critical to evaluate how these avatars perform across varying levels of health literacy, cultural backgrounds, and socioeconomic status to ensure the findings are generalizable beyond a tertiary care setting. Moreover, although this study was confined to plastic surgery, future research should extend to broader clinical contexts—particularly primary care and chronic disease management—where the scalability of AI avatars could play a pivotal role in enhancing longitudinal patient education and treatment adherence.

Beyond demographic considerations, more rigorous studies are needed to assess patients’ ability to detect deepfakes, comparing their self-reported confidence with their actual ability to distinguish authentic videos from manipulated ones. Additionally, experiments examining how patients perceive and respond to physician avatars delivering inaccurate or misleading information would help establish boundaries for avatar applications and identify trust-breaking thresholds.

To enhance the conversational ability of avatars, the next technical step is integrating retrieval-augmented large language models. This would enable generative yet accurate responses while reducing hallucinations [31,32]. More research is also needed on the effects of deepfake content warnings, as some participants in our study did not notice subtitles. This raises the question of whether patients may dismiss or overlook disclosure cues altogether [44].

Future studies should rigorously test whether media literacy interventions and active transparency signals (dynamic labels, voice cues, institutional markers) enhance acceptance, building on our finding that participants with prior deepfake exposure demonstrated higher engagement. Comparative studies should directly examine avatar effectiveness against alternative formats (cartoon characters, audio-only systems, text-based chatbots, or live video) within the same patient population to determine which embodiment best balances engagement, accessibility, and clinical utility. Long-term outcomes must also be explored, including information recall, adherence to medical advice, complication rates, and healthcare utilization.

### 4.7. Ethical Concerns

Integrating digital doctor twins into healthcare presents several ethical challenges. Accuracy is paramount: avatars must not disseminate misinformation or be vulnerable to tampering, which underscores the need for robust oversight to safeguard content [19]. Trust is equally critical. In our study, patients were aware that the avatar was synthetic, but a lack of disclosure or an unnatural delivery could erode trust not only in the avatar but also in the physician and the institution.

Developing avatars also requires careful attention to privacy and security, as the process involves sensitive biometric data such as facial features and voice patterns [54]. A critical concern is unauthorized impersonation: bad actors could create deepfake avatars of real physicians without consent, potentially spreading misinformation or exploiting patient trust. Robust authentication mechanisms and legal protections are essential to verify that clinical avatars are legitimate, physician-authorized representations. Healthcare institutions must establish clear frameworks distinguishing authorized clinical avatars from unauthorized synthetic media.

Creating a physician’s digital twin requires explicit, informed consent addressing several specific elements: (1) what the avatar will be used for (scope of clinical applications), (2) how long the avatar may be used, (3) whether the physician can withdraw or modify their likeness, and (4) who retains ownership and control of the digital representation. In our study, the surgeon provided consent for avatar creation and use in this research; however, clinical deployment will require standardized consent procedures that clearly communicate these dimensions to physicians before biometric data is collected [55].

A persistent challenge is governance of the digital likeness after a physician’s retirement, departure, or death. Key questions require institutional policy: Can the avatar continue operating after the physician leaves? Who decides whether the likeness may be archived, deactivated, or deleted? Can family members authorize continued use posthumously? Should there be a default expiration date for avatars? These governance gaps must be addressed through clear institutional protocols and potentially legislative frameworks before widespread clinical deployment.

The human element remains indispensable. There is risk that avatars could reduce genuine doctor–patient interaction if deployed as replacements rather than complements, particularly in emotionally sensitive discussions or complex decision-making where empathy is essential. Clear boundaries must define appropriate use cases (e.g., standardized postoperative FAQs) versus contexts requiring human clinician engagement (e.g., informed consent, bad news delivery).

Biases in training data or avatar design may perpetuate stereotypes and inequities [56]. Patient confusion about avatar capabilities could foster unrealistic expectations [57]. Finally, deepfakes can undermine trust in digital media more broadly. Phenomena such as the *Liar’s Dividend*, where even authentic video may be dismissed as fake, illustrate these wider societal risks [44]. Taken together, these concerns highlight the need for robust governance, full transparency, and strong ethical safeguards to ensure avatars are deployed responsibly in clinical care.

## 5. Conclusions

This feasibility pilot study provides preliminary evidence that patients demonstrate receptivity to hyper-realistic physician avatars for postoperative education. Among familiar patients in a controlled setting, the avatar achieved sufficient technical quality to engage patients without triggering significant discomfort or uncanny valley effects. These findings suggest that familiarity with the depicted physician and institutional credibility played key roles in facilitating acceptance, while transparency about the synthetic nature of the technology appeared to support rather than diminish trust.

The pilot demonstrates feasibility for further investigation. Specifically, we identified that avatar-based education can achieve high usability and engagement scores in targeted applications (standardized postoperative education). We also documented that patients can distinguish avatars from human clinicians while still trusting the medical information delivered, provided that the synthetic nature is disclosed. However, generalizability remains limited to similar settings (tertiary care, educated populations, familiar physician relationships) and requires validation in larger, more diverse populations and clinical contexts.

Future implementation of clinical avatars will depend on transparent, well-governed systems that clearly define appropriate use cases and maintain appropriate boundaries. Digital physician avatars may offer promise as a scalable tool for standardized patient education, but further research—including larger trials, diverse populations, long-term outcome assessment, and rigorous governance frameworks—is essential before broader clinical adoption. The human element of care remains irreplaceable; avatars are most appropriately deployed as educational complements, not replacements for human clinician engagement in complex or emotionally sensitive contexts.

## Figures and Tables

**Table 1:** Mean scores and standard deviations for patient evaluation of AI physician avatar across usability, engagement, trust, eeriness, and realism domains.

Metric	Item	Mean	SD
Usability	System Usability Scale (SUS)	87.67 (out of 100)	11.71
Engagement	Visually pleasing	4.47	0.57
	Absorbed in Interaction	4.3	0.6
	Enjoyable	4.27	0.58
	Worth time	4.43	0.63
	Rewarding	4.33	0.8
	Exciting	4.3	0.65
	Time slipped away	3.77	1.14
Acceptability & Trust	Information made sense	4.6	0.56
	Perceived as true	4.67	0.8
	From trusted source	4.5	0.57
	Trustworthy	4.6	0.5
	Will improve patient understanding	4.43	0.73
	Effective for education	4.47	0.68
	Would recommend to patients	4.6	0.56
	Believable information	4.7	0.47
	Overall Satisfaction	4.53	0.68
	Avatar matched past knowledge	3.9	1.21
Eeriness	Eerie, Strange, Unsettling	1.53	0.63
	Uncomfortable	1.4	0.5
	Mouth didn’t match	1.57	0.73
	Mouth moved strange	1.63	0.81
	Face Distorted, Uneven	1.73	0.91
Realism	Face looked stable	3.3	1.37
	Face looked clear	4.53	0.82
	Movement Stable	3.93	1.01
	Sound quality	4.73	0.52
	Voice sounded natural	4.37	1.13
	Voice match with physician	3.83	1.42
	To what extent did this agent seem like physician?	4.2	0.66
	Hard to tell if avatar was human or AI	2.7	1.12
	I would believe this was a real person	3.3	1.26

## Data Availability

The data collected for this study will be made available to others upon reasonable request. Access to the data will be granted to qualified researchers for non-commercial research purposes upon submission of a formal request and execution of a signed data access agreement. Any additional restrictions or conditions for data use will be clearly outlined in the data access agreement.

## Supplementary Materials

The following supporting information can be downloaded at: https://www.mdpi.com/article/10.3390/jcm14238595/s1.
