# Supplementary material for: Artificial Intelligence Physician Avatars for Patient Education: A Pilot Study
Source: J Clin Med. 2025 Dec 4;14(23):8595. doi: 10.3390/jcm14238595 (PMC12692777; doi:10.3390/jcm14238595)
Supplement: Supplementary file 1 [file jcm-14-08595-s001.zip › Supplementary material - Qualitative Analysis.pdf]

Major Themes Identified

THEME 1: COMMUNICATION EFFECTIVENESS

Agreed upon by all three researchers as the most prominent positive theme

Sub-themes:

- **Clarity and Comprehension** (n=8 mentions)
  - Representative quotes: "Easy to understand what he was saying," "Information explained in easy to understand language," "Clarity at verbage and pace"
  - *Researcher consensus: This sub-theme reflects the avatar's success in delivering comprehensible medical information*
- **Accessibility Benefits** (n=4 mentions)
  - Representative quotes: "Voice is easier/quicker to follow than if you were only reading," "Takes reading comprehension out of the process"
  - *Research team note: Particularly significant for patients with varying literacy levels*

Researcher Interpretation: All three researchers agreed this theme demonstrates the avatar's primary strength in overcoming traditional barriers to patient education, particularly for patients who struggle with written materials.

THEME 2: HUMAN-LIKE INTERACTION QUALITY

Researchers noted this theme's importance for patient engagement and trust

Sub-themes:

- **Realism and Believability** (n=6 mentions)
  - Representative quotes: "System looks very real as talking with real Dr," "Very believable as a credible source"
  - *Researcher B observation: High realism appears crucial for patient acceptance*
- **Personal Connection** (n=5 mentions)
  - Representative quotes: "Human like interaction to provide information more personal than handouts," "More engaging than written instructions"
  - *Researcher C note: Suggests avatar successfully bridges the gap between impersonal written materials and human interaction*

Research Team Consensus: The avatar's ability to create perceived human connection represents a significant advancement over traditional patient education modalities, with implications for patient satisfaction and information retention.

THEME 3: SYSTEM LIMITATIONS AND TECHNICAL ISSUES

All researchers identified this as the primary area requiring technical refinement

Sub-themes:

- **Movement and Visual Artifacts** (n=7 mentions)
  - Representative quotes: "Slight jilted movements at the end of questions," "Some small glitches with face," "Eyes a little unnatural"
  - *Researcher A observation: Technical imperfections may impact uncanny valley perceptions*
- **Audio Mismatches** (n=4 mentions)
  - Representative quotes: "Didn't sound like my surgeon," "Voice alignment" needed
  - *Research team debate: One participant viewed voice mismatch positively, suggesting individual preferences vary*

Researcher Interpretation: While technical limitations were noted, researchers agreed they did not fundamentally undermine system acceptance, suggesting users are tolerant of current deepfake technology limitations in healthcare contexts.

THEME 4: CONTENT SCOPE AND PERSONALIZATION

Researchers unanimously identified this as the most critical area for system development

Sub-themes:

- **Limited Question Range** (n=8 mentions)
  - Representative quotes: "Cannot answer all questions," "More questions need to be added," "Opportunity for more individualized questions"
  - *Researcher C emphasis: Reflects tension between standardized content and individual patient needs*
- **Lack of Procedure-Specific Information** (n=6 mentions)
  - Representative quotes: "Wasn't specific to my surgery," "More closely related to my procedure"
  - *Researcher B insight: Suggests need for modular, procedure-specific content architecture*

Research Team Analysis: All researchers agreed this theme represents the greatest barrier to widespread clinical implementation, requiring significant content development and personalization capabilities.

THEME 5: USABILITY AND ACCESSIBILITY

Researchers noted this as a key facilitator of system adoption

Sub-themes:

- **Ease of Use** (n=6 mentions)
  - Representative quotes: "Voice activated - no key strokes involved," "Simpleness"
  - *Researcher A note: Critical for elderly patient populations*
- **Immediate Access Benefits** (n=4 mentions)
  - Representative quotes: "You receive immediate answers," "Don't have to wait for someone to respond"
  - *Research team consensus: Addresses healthcare access and timing challenges*

Researcher Interpretation: High usability scores suggest the system successfully meets patient expectations for intuitive healthcare technology interaction.

Researcher-Specific Observations

Researcher A Focus: Technical Implementation

- Emphasized the significance of visual artifacts in potentially triggering uncanny valley responses
- Noted the importance of addressing technical limitations before clinical deployment
- Highlighted positive correlation between technical quality and trust metrics

Researcher B Focus: Clinical Integration

- Stressed the need for procedure-specific content development
- Identified potential workflow integration challenges
- Emphasized importance of maintaining clinical accuracy while expanding content

Researcher C Focus: Patient Experience

- Highlighted the emotional and psychological benefits of human-like interaction
- Noted individual variation in technology acceptance and preferences
- Emphasized accessibility benefits for diverse patient populations

Collaborative Improvement Framework

Through consensus, the research team developed the following prioritized improvement categories:

High Priority (Unanimous Agreement)

1. Content expansion and personalization capabilities
2. Technical refinement of visual rendering
3. Procedure-specific information architecture

Medium Priority (2/3 Researcher Agreement)

1. Enhanced interaction modalities (typing, multi-part questions)
2. Voice synthesis improvements
3. Integration with healthcare delivery systems

Low Priority (Individual Researcher Preferences)

1. Multi-language support
2. Multi-gender avatar options
3. Advanced visual aids and demonstrations
